# Eosinophilia in critically ill COVID-19 patients: a French monocenter retrospective study

**DOI:** 10.1186/s13054-020-03361-z

**Published:** 2020-11-03

**Authors:** Megan Fraissé, Elsa Logre, Hervé Mentec, Radj Cally, Gaëtan Plantefève, Damien Contou

**Affiliations:** Service de Réanimation Polyvalente, Centre Hospitalier Victor Dupouy, 69, rue du Lieutenant-Colonel Prud’hon, 95100 Argenteuil, France

## Introduction

As reported in bacterial sepsis [1], the early phase of SARS-CoV-2 infection seems to be accompanied by eosinopenia [2–4]. Conversely, our team noticed that several of our critically ill COVID-19 patients developed unexpected and unexplained eosinophilia during their ICU stay. Indeed, as white blood cells count is performed almost daily, monitoring and studying eosinophil course is simple in the ICU setting. To our knowledge, no study has focused on eosinophilia in COVID-19 although eosinophil recovery seven days after initial eosinopenia seems to be associated with a better outcome [3].

We aimed to assess the incidence and to describe eosinophilia in critically ill COVID-19 patients, as well as to compare the outcome between patients developing or not eosinophilia during their ICU stay.

## Methods

We retrospectively reviewed all daily white blood cells counts performed in adult COVID-19 patients (RT-PCR positive for SARS-CoV-2) admitted to our 40-bed COVID-19 ICU between March 6 and July 30, 2020. Eosinophilia was defined by an eosinophil count higher than 500/mm^3^ and was considered as severe when exceeding 1500/mm^3^. Eosinopenia was defined by an eosinophil count lower than 40/mm^3^.

## Results

During the study period, 99 patients were admitted for acute respiratory failure related to SARS-CoV-2 pneumonia. After excluding 21 patients transferred to another ICU, 78 patients remained in the analysis. Among them, 69 (88%) had eosinopenia at ICU admission and 26 (33%, 95% confidence interval 23–45%) developed eosinophilia during ICU stay.

Among the 26 patients who developed eosinophilia (Table 1), 22 (85%) had eosinopenia at ICU admission. Eosinophilia occurred 19 [13–28] days after ICU admission and lasted 5 [3–12] days. Median eosinophil count was 900 [678–1350]/mm^3^. Six patients (23%) developed severe eosinophilia. Seven patients (29%) had a biphasic eosinophilia. Ten (38%) patients were treated with a β-lactam antibiotic when eosinophilia occurred. Eleven (42%) patients had at least once a temperature > 38.3 °C and 2 (8%) had an erythematous skin rash during the eosinophilia period, respectively.

**Table 1:** Description of eosinophilia (> 500/mm^3^) in 26 critically ill patients with SARS-CoV-2 pneumonia

	Patients with eosinophilia during ICU stay *N* = 26
Eosinophil count at ICU admission	
Median eosinophils count (cell/mm^3^)	5 [0–20]
Eosinophilia (> 500/mm^3^)	1 (4)
Eosinopenia (< 40/mm^3^)	22 (85)
No eosinophil	13 (50)
Normal eosinophil count	3 (11)
Eosinophilia during ICU stay	
Days between ICU admission and eosinophilia	19 [13–28]
Days between disease onset and eosinophilia	30 [23–38]
Days between disease onset and pic of eosinophilia	31 [25–42]
Median eosinophil count (cells/mm^3^)	900 [678–1350]
Severe eosinophilia (> 1500/mm^3^)	6 (23)
Total duration of eosinophilia (days)	5 [3–12]
Biphasic eosinophilia	7 (27)
Administration of β-lactam antibiotics during eosinophilia period	10 (38)
Temperature > 38.3 °C at least once during eosinophilia period	11 (42)
Erythematous skin rash during the eosinophilia period	2 (8)
Treatment with ivermectin for eosinophilia	3 (12)

Continuous variables are reported as median [Interquartile range] and categorical variables are reported as numbers (percentage)

Comparison between patients with and without eosinophilia during ICU stay is detailed in Table 2.

**Table 2:** Comparison between 78 critically ill patients with SARS-CoV-2 pneumonia developing (*n* = 26) or not (*n* = 52) eosinophilia (eosinophils count > 500mm^3^) during ICU stay

	All patients *N* = 78	Patients with eosinophilia *N* = 26	Patients without eosinophilia *N* = 52	*p*
Patients characteristics and ICU scores				
Male sex	62 (79)	22 (85)	40 (77)	0.62
Age, years	62 [54–70]	62 [55–70]	62 [54–70]	0.84
SOFA	4 [3–7]	4 [3–7]	4 [3–8]	0.44
SAPS II	33 [22–44]	34 [28–38]	31 [19–44]	0.72
Days between disease onset and ICU admission	8 [7–12]	10 [7–13]	8 [7–12]	0.28
Main comorbidities, *n* (%)				
Obesity (body mass index ≥ 30 kg/m^2^)	36 (46)	9 (36)	27 (53)	0.25
Arterial hypertension	51 (65)	13 (50)	38 (73)	0.08
Diabetes mellitus	33 (42)	12 (46)	21 (40)	0.81
Ischemic cardiopathy	8 (10)	3 (12)	5 (10)	1.00
Cerebro-vascular diseases	7 (9)	4 (15)	3 (6)	0.21
Venous thrombo-embolism	5 (6)	2 (8)	3 (6)	1.00
Chronic respiratory diseases	18 (23)	6 (24)	12 (24)	1.00
Chronic renal failure	7 (9)	2 (8)	5 (10)	1.00
Recent cancer or hemopathy	3 (4)	1 (4)	2 (4)	1.00
ACE or ARB	35 (45)	11 (42)	24 (47)	0.88
Biological data at ICU admission				
Median eosinophils count (cell/mm^3^)	0 [0–10]	5 [0–20]	0 [0–10]	0.43
Eosinophilia (> 500/mm^3^)	1 (1)	1 (4)	0 (0)	0.33
Eosinopenia (< 40/mm^3^)	69 (88)	22 (85)	47 (90)	0.47
No eosinophils	47 (60)	13 (50)	34 (65)	0.29
Fibrinogen (g/L)	8 [6–9]	7 [6–8]	8 [7–9]	0.37
D-dimers (µg/mL)	2440 [1570–9915]	2415 [1968–13670]	2720 [1400–7250]	0.36
Prothrombin time (%)	85 [75–96]	87 [80–96]	85 [70–96]	0.59
Platelets count (G/L)	225 [164–291]	209 [190–327]	226 [161–272]	0.34
Treatment for SARSCoV-2 pneumonia				
Glucocorticoids^a^	12 (15)	4 (15)	8 (15)	1.00
Hydroxychloroquine^b^	2 (3)	1 (4)	1 (2)	1.00
Azithromycin	0	–	–	–
Remdesivir	0	–	–	–
Lopinavir–ritonavir	0	–	–	–
Tocilizumab	0	–	–	–
Outcomes in the ICU				
Invasive mechanical ventilation	68 (87)	25 (96)	43 (83)	0.15
Prone positioning	48 (62)	18 (69)	30 (58)	0.46
Vasopressor support	49 (63)	19 (73)	30 (58)	0.28
Acute kidney failure	55 (71)	19 (79)	36 (69)	0.53
Renal replacement therapy	24 (31)	12 (46)	12 (23)	0.07
Ventilator-associated pneumonia	45 (58)	19 (73)	26 (50)	0.09
Thrombotic event during ICU stay	33 (42)	12 (48)	21 (40)	0.70
Length of ICU stay, days	16 [8–30]	31 [23–52]	12 [6–21]	< 0.001
ICU mortality	38 (49)	9 (35)	29 (56)	0.13
Days between ICU admission and death	13 (8–21)	23 (18–47)	12 (8–16)	< 0.001

Continuous variables are reported as median [Interquartile range] and compared between groups using the Student *t* test. Categorical variables are reported as numbers and percentages and compared using *χ*^2^ test. A *p* value < 0.05 was considered significant

*ACE/ARB* angiotensin-converting enzyme inhibitors/angiotensin receptor blockers, *ICU* intensive care unit, *SAPS2* simplified acute physiology score, *SOFA* sepsis-related organ failure assessment

^a^In a context of randomized clinical trial (*n* = 10) or as a salvage therapy (*n* = 2)

^b^In a context of randomized clinical trial

By using a Cox model with time-varying covariate and after adjustment for SAPSII, age and administration of glucocorticoids, eosinophilia was associated with a decreased ICU mortality (HR = 0.44, 95% CI 0.23–0.85, *p* = 0.014).

## Discussion

Despite an 88% rate of eosinopenia at ICU admission, we report that one-third of our critically ill COVID-19 patients developed an unexpected late-onset and prolonged ICU-acquired eosinophilia which was severe in almost one quarter of them. Such a high rate of eosinophilia is uncommon in non-COVID-19 critically ill patients and has never been documented in other viral infection such as influenza. A clear explanation for eosinophilia was not retrieved in our patients even if a drug-induced eosinophilia could not be formally ruled out. Some patients with severe eosinophilia were even empirically treated with ivermectin for a hypothetic helminthiasis-related eosinophilia. Given that eosinophilia was a late-onset event in the course of ICU stay, its positive impact on survival is difficult to interpret, patients developing eosinophilia being exposed to a survival bias.

Considering the absence of a clear explanation for the high rate of eosinophilia observed in our patients, we can legitimately hypothesize that SARS-CoV-2 was directly or indirectly responsible for eosinophilia, as a consequence of infection or recovery. The late occurrence of eosinophilia is consistent with the prolonged SARS-CoV-2 RNAaemia reported in critically ill patients [5]. Whether eosinophilia is a marker of an excessive immune recovery or a dysregulated immune response during the cytokine storm [6] induced by the infection is unknown.

Even if our study suffers from several limitations, our findings emphasize the underestimated and understudied role of eosinophils in COVID-19. Further, larger studies are needed to overcome these limitations.

## Data Availability

The dataset used and analyzed for the current study is available from the corresponding author on reasonable request.
